# Erratum: comparison of the sagittal profiles among thoracic idiopathic scoliosis patients with different Cobb angles and growth potentials

**DOI:** 10.1186/s13018-014-0082-7

**Published:** 2014-10-24

**Authors:** Bo Ran, Xiang-yang Chen, Guo-you Zhang, Feng Shen, Jia-yu Chen, Ji-bin Wu, Feng-chao Zhao, Dun-yi Qiao, Bing Zhou, Xin-zhu Zhang, Yue-hua Qiao, Jun-hui Guan, Kai-jin Guo, Ming Li

**Affiliations:** Department of Orthopedics, Xuzhou Medical College Affiliated Hospital, Xuzhou Medical College, Xuzhou, 221006 China; Department of Orthopedics, Changhai Hospital Affiliated to Second Military Medical University, Shanghai, 200433 China; Department of Orthopedics, Kunming General Hospital of Chengdu Military Command, Kunming, 650032 China; Taizhou Municipal Hospital, Taizhou, Zhejiang Province 318000 China

## Correction

After publication of this work [1], we noted that we inadvertently failed to include the complete list of all co-authors, and they were not listed in the correct order. In addition, we have provided updated Authors’ Contributions and Competing interests sections. We apologize for any inconvenience these oversights may have caused.
